# Storytellers as partners in developing a genetics education resource for health professionals

**DOI:** 10.1016/j.nedt.2011.11.019

**Published:** 2013-05

**Authors:** Maggie Kirk, Emma Tonkin, Heather Skirton, Kevin McDonald, Buddug Cope, Rhian Morgan

**Affiliations:** a NHS National Genetics Education and Development Centre, University of Glamorgan, Pontypridd, Wales, United Kingdom; b Faculty of Health and Social Work, University of Plymouth, Wellington Road, Taunton, Somerset, England, United Kingdom; c Genomics Policy Unit, Faculty of Health, Science and Sport, University of Glamorgan, Pontypridd, Wales, United Kingdom; d Genetic Alliance UK, Wales Gene Park, Heath Park, Cardiff, United Kingdom

**Keywords:** Genetics, Genomics, Health professional, Education, Stories, Storytelling

## Abstract

Advances in genetics are bringing unprecedented opportunities for understanding health and disease, developing new therapies and changes in healthcare practice. Many nurses and midwives lack competence and confidence in integrating genetics into professional practice.

One approach to enhance understanding of genetics is to simulate clinical exposure through storytelling. Stories are acknowledged as a powerful learning tool, being understandable and memorable, stimulating critical thinking, and linking theory to practice. Telling Stories, Understanding Real Life Genetics is a freely accessible website that sets people's stories within an education framework. The links between the stories and professional practice are made explicit and additional features support learning and teaching.

Care of the storytellers within an ethical framework is of paramount importance. Storytellers are viewed as partners in the project. The challenges encountered include preserving the authentic voice and dignity of the storyteller. Project team members have also experienced ‘professional shame’ when negative experiences have been recounted, and the stories have had an impact on the team.

The experience of working with storytellers has been positive. The storytellers want to be heard so that others will benefit from their stories. They serve as a reminder of why this work is important.

## Introduction

Helen went swimming one morning with her daughter and son, an apparently healthy, fit 19 year-old. He swam to the end of the pool, collapsed and died. Helen's story tells of her determination to ‘make sense’ of her son's death and to identify the cause. It was eventually discovered that he died from an inherited cardiac condition (Long QT syndrome), and that her husband, daughter and grandchildren are also at risk from the condition, for which they are now receiving treatment. There was a very strong family history of sudden cardiac death, which had never been picked up by health professionals. Helen told her story because she wanted to raise awareness about Long QT.

*I was so often dismissed as being a neurotic mother and I'm not a neurotic mother. … I love my children and I love my husband and I didn't want it happening again and that's the reason I am doing this now …*

Helen (www.tellingstories.nhs.uk)

Helen's account is from a collection of stories gathered to provide a web-based education resource for health professional groups, to promote awareness and understanding about genetics and how it impacts on people's lives. Advances in genetics are bringing unprecedented opportunities for understanding health and disease, developing new therapies and changes in healthcare practice (Green and Guyer, 2011). Amongst the key achievements noted by the House of Lords Science and Technology inquiry into genomic medicine (2009) are:

• Predictive diagnosis and single gene conditions, with genetic tests for over 1000 diseases currently available for clinical testing.
• Diagnosis of genetic subtypes of common diseases such as diabetes, Alzheimer's disease, Parkinson's disease and several types of cancer.
• Use of genetic-genomic tests to inform disease management, e.g. through tumour profiling to identify breast cancer patients who are more likely to respond to trastuzumab (Herceptin).
• Predicting individual responsiveness and side effects to certain drugs, e.g. responsiveness to warfarin, or to identify potential hypersensitivity reaction to abacavir for HIV treatment.

This paper will outline the value of stories in education and how they have been used in the development of a genetics education resource for healthcare professionals. It will also discuss how the storytellers have been involved as partners in the process.

### Background

Calls for health professionals working outside of specialist genetics services to be better educated in genetics-genomics are longstanding and even in countries with established strategies for development, there is an acknowledgement of the scale of the challenge (Department of Health, 2008; Green and Guyer, 2011). There is a substantial body of evidence to show that there are significant deficits in genetics education of nurses nationally and internationally, with no real foundation of knowledge on which to build (for a review see Burke and Kirk, 2006). Many UK nurses and midwives have difficulty in making a connection between genetics and their professional practice, and many lack confidence in integrating genetics knowledge and skills into practice (Kirk et al., 2007; Metcalfe et al., 2007), a finding also highlighted in a review of genetic competence of midwives (Skirton et al., 2010). This is compounded by educators who lack confidence in teaching genetics, and who may have limited clinical experience of genetics (Kirk and Tonkin, 2006). In a global survey of nurse leaders, participants identified a lack of professional engagement in genetics–genomics as a result of inadequate awareness and knowledge among educators and practitioners amongst the significant barriers to fully integrating genetics–genomics into nursing education (Kirk et al., 2011a).

In their review, Tonkin et al. (2011) note the growing abundance of genomic resources for nurse education but acknowledge that finding the most appropriate resource can be taxing. A survey of nurse educators (Kirk and Tonkin, 2006) reported the three highest ranked resources needed to support genetics teaching as:

• Access to users or providers of genetics services willing to talk to student groups;
• Annotated scenarios and case studies;
• Websites.

With access to genetics service users constrained by geography and by pressure to limit observers at genetics clinics to specialist trainees, opportunities for ‘clinical exposure’ are limited. One approach to enhancing nurses' understanding of genetics is to simulate clinical exposure through the stories of people affected by genetics.

### Stories in Healthcare Education

The development of web sites dedicated to patient stories is a relatively recent phenomenon (e.g. Healthtalkonline, www.healthtalkonline.org; Patient Voices, www.patientvoices.org.uk/) but storytelling is recognised as a valuable resource within healthcare, with a ‘growing realisation that patients and service users are a rich source of health-care related stories that can affect, change and benefit clinical practice’ (Haigh and Hardy, 2010; p411). Wilcock et al. (2003) argue the potential of patient storytelling to inform improvement in healthcare by responding to the needs of patients and carers Ziebland and Herxheimer (2008) echo this, calling for health professionals to recognise the value of patient stories as a contribution to the evidence base, particularly in capturing and communicating the essence of patient experience. Gregory (2010; p630) states that ‘patients are no longer regarded as passive recipients of healthcare; they have become active participants, with personal stories to tell about the journeys they have travelled from sickness to health.’ She reflects on how narratives can provide insight into the patient's perception of their own therapeutic and rehabilitation needs and, in conveying patient experiences, how they can encourage healthcare professionals to reflect on their practice and respond to service-user needs. Kirk et al. found that a ‘lack of attention paid to the patient voice’ (Kirk et al., 2011a; p110) was cited by nurse leaders as a significant barrier to the integration of genetics-genomics into nursing education.

Stories are acknowledged as a powerful learning tool in health professional education (e.g. Christiansen, 2011; Schwartz and Abbott, 2007). The literature suggests that stories can promote learning because they:

• attract the attention of the student and draw them into the world of another (Christiansen, 2011)
• are understandable and memorable (Cox, 2001).
• stimulate critical thinking (Kirkpatrick et al., 1997)
• can help link theory and practice (Koenig and Zorn, 2002)

Davidson (2004), examining an education approach that used storytelling as a primary tool, found it provided opportunities for more active involvement of students and made the material seem more realistic. Lordly (2007) found a similar positive effect in the classroom, and concluded that stories can influence how students approach professional practice. Storytelling has also been reported to benefit midwifery students by increasing their cognitive skills, developing their emotional skills and by helping to define their role (Hunter and Hunter, 2006). Its merit in helping to develop empathy, explore ethical issues and promote tolerance and cultural sensitivity amongst healthcare providers is also recognized (Fairbairn 2002). Cangelosi and Whitt (2006) suggest that storytelling in an online learning environment is an effective and efficient education approach, helping students learn through sharing, reflection and interpretation of stories. The literature thus seemed to support our intention to develop an online genetics education resource using stories.

## Telling Stories Understanding Real Life Genetics

**Fig. 1:**
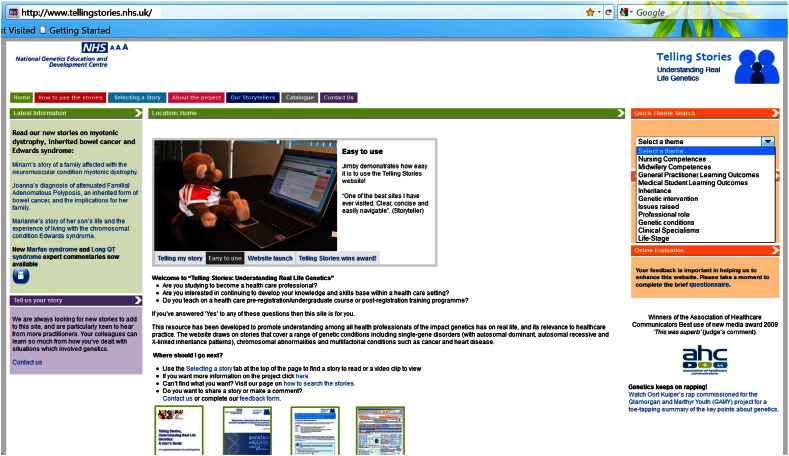
Home screen of the resource website. Menu tabs facilitate navigation and use of site. Search via selected themes is available through a drop-down menu (right hand box).

**Fig. 2:**
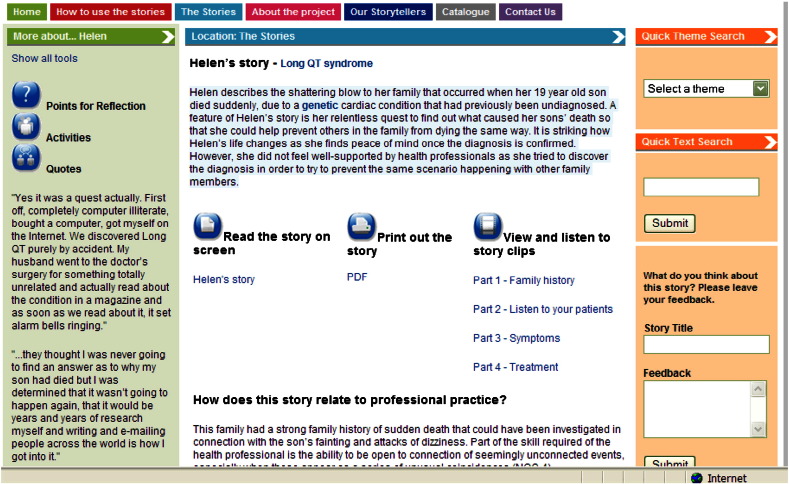
Screen capture of the Telling Stories website. The figure shows: that the stories are accompanied by additional resources to support teaching and learning (left-hand section); that the stories are available to read on screen, or downloaded as a PDF file, accompanied by video clips, and make explicit the link to professional practice (middle section) the site is searchable according to theme or key word (right-hand section). There is a facility for online feedback.

*Telling Stories, Understanding Real Life Genetics* is a website of over 100 stories, hosted by the NHS National Genetics Education and Development Centre (Fig. 1; www.tellingstories.nhs.uk). The stories (available as text supported with video clips) are organised into 11 themes to aid searching and lesson planning: professional competences and learning outcomes illustrated; genetic condition; inheritance pattern; genetic intervention; professional role; issues raised; clinical specialism; life-stage. The resource is enhanced by additional features (Fig. 2) including suggested activities to accompany learning or teaching, and explanatory notes on how the story links to professional practice via the UK genetics-genomics education framework (Kirk et al., 2003, 2011b).

There were three key considerations in the development of the resource.

### Educational Value and Accuracy of Content

While stories are compelling in their own right, the main function of the website is educational and its development is underpinned by adult learning principles (Knowles et al. 2005). Links between a story and professional practice are made explicit and users are encouraged to draw and reflect on their own experience. It is imperative that the content is accurate and that the stories are supported by information that enables users to develop their knowledge base. To support self-directed learning, we aim to make the site accessible to the novice, while facilitating further learning beyond the actual stories. Key terms within the story are identified and included in a glossary. Basic information about the condition is added to each page, including the inheritance pattern, signs and symptoms of the condition and medical management. Given the many thousands of genetic conditions, it is important that we take this approach, and we direct users to reliable and appropriate external resources, to seek further information about genetic conditions for themselves and their patients.

Ensuring accuracy of the content is fundamental. To ensure a high standard, the project team ensures that each story is assessed and annotated by a subject expert. A first annotation is undertaken by a team member, and all information on the site is checked by one team member with expertise in clinical genetics.

One of the challenges in developing and maintaining the resource is to preserve the authentic voice and dignity of the storyteller, potentially compromised for example where scientific misunderstandings might be presented as fact. This has sometimes created a dilemma. Philosophically the project is about the experience of the story tellers and to change their words is inappropriate. However, leaving inaccuracies in the text in an educational site is not acceptable. The team has compromised by placing a note next to any phrases that are not accurate, leading the reader to the correct information.

### Effective and Informed Application of Technology

The design of the pilot website evolved in parallel with the development of the resource content and was informed by principles of good web architecture and design. The aim was to create an online, interactive, multi-media educational resource to support health professionals understanding of genetics through collaborative input from members of the project team with expertise in genetics and genetic counselling, health professional education, research and information technology.

Key considerations for the technological development of the website were that it should:

• Be clear and easily navigable
• Provide an easy to use multi-themed search facility and a content catalogue
• Give options to guide new visitors on how to use the web content through a logical sequence and to direct experienced users straight to the specified content
• Create a digital environment with accessible and (re)useable content in ‘bite-size chunks’
• Cater for a range of healthcare professionals
• Offer opportunities for blended learning
• Facilitate user feedback
• Enable future expansion
• Ensure the provision of secure web data
• Generate statistics on website usage.

Consequently, through a combination of efficient programming techniques, appropriate server and database back-end selection, and effective architecture, we developed a website that incorporated all of the desired features. This has been reflected by user endorsements.

‘One of the best sites I have ever visited. Clear, concise and easily navigable’.

Storyteller

### Working with Storytellers

Care of the storytellers within an ethical framework, ensuring informed consent, privacy, anonymity and respect, is of paramount importance and storytellers and their representatives are viewed as partners in the project. This is reflected at all stages from project team membership and planning, to evaluating the resource, participation in its launch, and in raising awareness about the website.

One member of the team (BC) is a member of an umbrella charitable organisation, Genetic Alliance UK, which represents the interests of all people with or at risk of a genetic condition. This is a valuable factor in gaining access to support groups through Genetic Alliance UK's extensive network, and in disseminating news about the project.

Following ethics committee approval, storytellers are recruited via a variety of means, including through newsletters and talks at conferences for members of voluntary support groups. Those expressing an interest in becoming involved are provided with an information sheet and contact details for the project officer. She establishes contact with potential storytellers and ensures that they understand the aims and process of the project. When individuals are ready, they are asked to sign the consent and copyright release forms for the story to be published on the website and through other dissemination material. Stories are gathered either in text format only, or via video-recording. The names of storytellers are altered by the project officer, who takes care to assign ethnically appropriate pseudonyms. Other identifying details (such as names of hospitals or health professionals) are removed from text-based stories and, as far as possible, by careful editing of video clips.

People submitting written stories are given 3–4 open questions to guide their narrative. Interviews follow a similar format, with interviewees being invited to talk about the particular genetic condition, how it affects them and their family, and about their related healthcare experiences. Once the stories have been transcribed and/or prepared for the website, storytellers are approached to provide final consent. They are also invited to check the story once it appears on the website, and can ask for the story to be removed at any time. At this juncture, storytellers are invited to complete an evaluation form which includes questions about their experience of being a storyteller and their opinion of the completed story.

Paul's experience of participating in the project echoes those of other storytellers, and their motivation to become involved:

*No pressure was put on me to include sensational bits or to include family members who might be concerned. At the end I felt worn out and yet really pleased at being given the opportunity to be part of a new way of training where patients’ stories would really be appreciated and help fill in the gaps that text books are unable to do.*

Paul (www.tellingstories.nhs.uk)

**Fig. 3:**
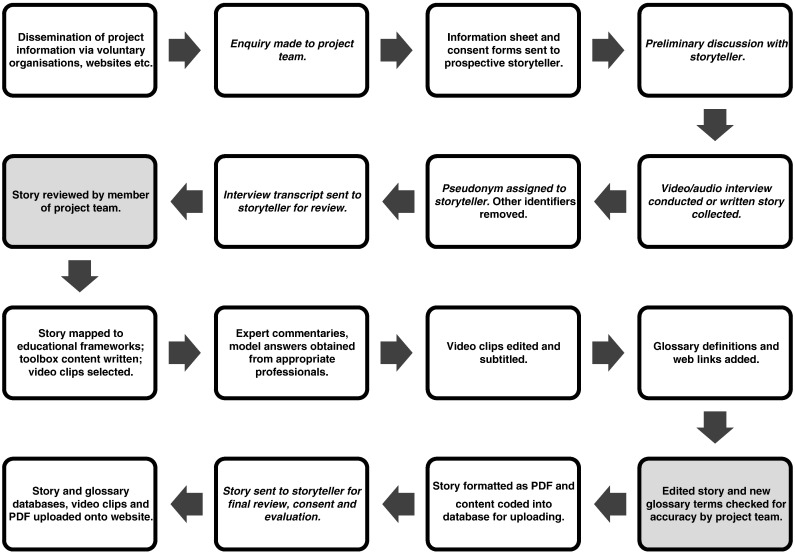
Overview of resource development process. The process begins with the dissemination of project information to potential participants (top left) through to publication of the story and accompanying educational material on the website (bottom left). Text in italics denotes the stages where the storyteller is involved directly. Shaded boxes indicate where content is reviewed and validated by the project team. Storytellers have the option to withdraw their story at any time, even after website publication.

The project team remain in touch with many storytellers and we value their contribution to ongoing dissemination about the site. An overview of the process by which we work in partnership with the storytellers to collect and develop their stories as an educational resource for the website is shown (Fig. 3).

The resource is freely available and all material can be reproduced for education purposes. However, visitors are asked to ensure its use is consistent with the ethos of the project and to maintain respect for the storytellers.

## Evaluating the Resource

The evaluation strategy for the resource is comprehensive and ongoing. Prior to the site going ‘live’ it was reviewed by the Centre's network of nurse educators and two e-learning specialists. Involving the storytellers at this stage was important, and a total of 26 out of 30 storytellers provided feedback on style, navigability and accuracy. Feedback included:

*We feel that this website could make an important difference to the training of professionals and its application and use should be widened to include doctors at ALL levels* (21).

*I think that for me, it was a form of therapy — I needed to ‘shout out’ and it's taken me 24 years! Thanks for giving me that release!* (01).

The Telling Stories website was launched in June 2007, and delegates were asked for their feedback on the style and content of the website, their confidence in using it and their intended use of the resource. One launch delegate stated:

*Seeing the videos was the most positive aspect because it grounded my learning as not just textbooks but human experience* (L27).

Follow-up evaluation was conducted three to four months later to explore healthcare educators' longer-term views on its relevance and usefulness (Burke 2008). Participants were asked about their motivation for attending the launch, website use and accessibility, their views on using patient stories as an educational tool, dissemination of information about Telling Stories, its impact on their own learning and teaching practice, and suggestions for future development. Comments included:

*Every time you read it you think of some other aspect that you might not have thought of… I think it's particularly useful because in genetics… it can be quite a while between you seeing one condition and you seeing another (Ms B).*

*It's real people… these are individuals that have this genetic disorder. And as much as it's important for [health professionals] to know the inheritance patterns and the symptoms and the sites of symptoms, they also need to know how it affects the whole person. I think the Telling Stories website provides this resource in a very, very meaningful way (Ms G).*

This feedback was encouraging with educators stating that the website was relevant, easy to navigate and use, had impacted on their own learning, and that they had developed specific plans to use it in future teaching. Other positive aspects included the value of using authentic patient stories within education, the opportunity to learn from expert patients/families, use of the stories to link theory and practice, that ethical approval for using the stories had already been granted and that the stories are available in multiple formats. Barriers to the use of the resource included a lack of time, both in terms the limited time devoted to genetics in teaching curricula and educator workload, and potential technological barriers.

**Fig. 4:**
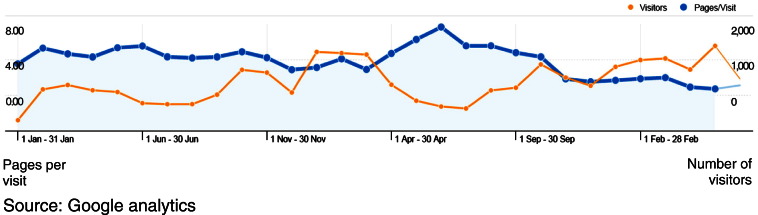
Site visitors and pages per visit January 2010–June 2011.

During its first 12 months, the website received 105,097 hits to specific pages, and 21,401 downloads. These have originated from all continents except Antarctica. Since January 2009, Google Analytics software (http://www.google.com/analytics/) has been used to monitor and evaluate web statistics (Fig. 4). Since this time to the present, the website has received over 33,100 visits with approximately 167,000 page views from over 150 countries/territories worldwide. These data are being utilised to understand how individuals are using the site, inform site development in order to reflect the changing needs of users and inform strategies to continue to promote the site amongst the intended target audiences (Kirk et al., 2008). This particular aspect of the resource will be the subject of a separate paper.

Visitors to the website are invited to participate in an online evaluation questionnaire which includes questions about their experience of using the website. Users are also invited to provide comments or feedback about all aspects of the website, such as the stories themselves or technical issues, via a dedicated webpage.

Evaluation of the resource using storyteller and user feedback has been integral to informing its continual development and improvement. Examples of this include adding more stories and new searchable themes, inclusion of genetics education frameworks for other healthcare professionals, addition of expert commentaries from specialist professionals, and provision of downloadable user guides (for example, Frequently Asked Questions guide).

### Reflection

Many of the stories collected so far demonstrate the courage and resilience of the storytellers, coping with sometimes extremely difficult circumstances. In her research, Christiansen (2011) found that for some students, the patient digital story could be unsettling. Project team members experienced ‘professional shame’ when negative experiences were recounted, and these stories had an impact on us. Sickle cell disease (SCD) is a recessively-inherited disorder characterised by periodic bouts of crisis when red blood cells assume a distorted (sickle) shape because of reduced oxygen-binding capacity. These crises are associated with excruciating pain, as Patrick describes:

*When the pain…the worst thing is your chest. It feels as if it's squeezing your chest, squeezing. When you are lying down you are restless because you try to turn in all different positions thinking it would ease the pain… and two minutes after it starts up again worse.*

Patrick (www.tellingstories.nhs.uk)

The impact of a lack of awareness of SCD is revealed in Tony's experience:

*…I was in hospital and I just wanted to cry really. Because the ward they put me on, the staff didn't know anything about it. Not a bit. I had to argue with one staff who gave me paracetamols … and like I said I had to argue, literally argue…*

*That's one of the worst experiences I have ever had and to me the most frightening. Like I said, now I'm scared of going back into hospital….*

*A lot of people still don't understand. That's the main thing that gets me. They still do not understand in this day and age. And they don't believe you. Unless you've dealt with that patient constantly nobody believes that you can be in that much pain… As long as you get some sort of educational understanding about it that would please me.*

Tony (www.tellingstories.nhs.uk)

Hearing a mature adult talk of his fear of hospital admission because of the anticipation of suffering was unsettling, but the positive intent of the storytellers to improve care for others is heartening. Other stories illustrate the value of good professional care. Maria did not expect her health visitor to be expert in genetics, and clearly appreciated an open, positive attitude:

*It was very fortunate for us that we had a brilliant Health Visitor who gave us all the support she could offer. She helped us find out all that she could, even though she knew as little as we did. She spent much of her own time doing research on the internet about the condition and where to get some special growth charts for my son to record his progress.*

Maria (www.tellingstories.nhs.uk)

## Conclusion

The resource created is unique in linking to education frameworks, and feedback indicates that it is welcomed within the UK and internationally. The experience of working with storytellers has been overwhelmingly positive. One clear message is that the storytellers want to be heard so that others will benefit from their stories. They serve as a reminder of why this work is important.

## References

[bb0005] Burke S. (2008). Telling Stories, Understanding Real Life Genetics. Educators’ perceptions of relevance and usefulness: Preliminary Report.

[bb0010] Burke S., Kirk M. (2006). Genetics education in the nursing professions: a literature review. Journal of Advanced Nursing.

[bb0015] Cangelosi P.R., Whitt K.J. (2006). Teaching through storytelling: an exemplar. International Journal of Nursing Education Scholarship.

[bb0020] Christiansen A. (2011). Storytelling and professional learning: a phenomenographic study of students' experience of patient digital stories in nurse education. Nurse Education Today.

[bb0025] Cox K. (2001). Stories as case knowledge: case knowledge as stories. Medical Education.

[bb0030] Davidson M.R. (2004). A phenomenological evaluation: using storytelling as a primary teaching method. Nurse Education in Practice.

[bb0035] Department of Health (2008). Our inheritance, Our Future - Realising the potential of genetics in the NHS. Progress review.

[bb0040] Fairbairn G. (2002). Ethics, empathy and storytelling in professional development. Learning in Health and Social Care.

[bb0045] Green E.D., Guyer M.S., National Human Genome Research Institute (2011). Charting a course for genomic medicine from base pairs to bedside. Nature.

[bb0050] Gregory S. (2010). Narrative approaches to healthcare research. International Journal of Therapy and Rehabilitation.

[bb0055] Haigh C., Hardy P. (2010). Tell me a story — a conceptual explanation of storytelling in healthcare education. Nurse Education Today.

[bb0060] House of Lords Science and Technology Committee (2009).

[bb0065] Hunter L.P., Hunter L.A. (2006). Storytelling as an educational strategy for midwifery students. Journal of Midwifery & Women's Health.

[bb0090] Kirk M., Tonkin E. (2006). Genetics education for nursing professional groups: survey of practice and needs of UK educators in delivering a genetics competence framework.

[bb0095] Kirk M., McDonald K., Longley M., Anstey S. (2003). Fit for practice in the genetics era: a competence based education framework for nurses, midwives and health visitors. Final Report to Department of Health.

[bb0085] Kirk M., Tonkin E., Birmingham K. (2007). Working with publishers: a novel approach to ascertaining practitioners’ needs in genetics education. Journal of Research in Nursing.

[bb0080] Kirk M., Tonkin E., Burke S. (2008). Engaging nurses in genetics: The strategic approach of the NHS National Genetics Education and Development Centre. Journal of Genetic Counselling.

[bb0070] Kirk M., Calzone K., Arimori N., Tonkin E. (2011). Genetics-Genomics Competencies and Nursing Regulation. Journal of Nursing Scholarship.

[bb0075] Kirk M., Tonkin E., Skirton H. (2011). Fit for Practice in the Genetics/Genomics Era: a revised competence based framework with Learning Outcomes and Practice Indicators. A guide for nurse education and training.

[bb0100] Kirkpatrick M.K., Ford S., Castelloe B.P. (1997). Storytelling: an approach to client-centred care. Nurse Educator.

[bb0105] Knowles M.S., Holton E.F., Swanson R.A. (2005). The adult learner, the definitive classic in adult education.

[bb0110] Koenig J.M., Zorn C.R. (2002). Using storytelling as an approach to teaching and learning with diverse students. Journal of Nursing Education.

[bb0150] Lordly D. (2007). Once upon a time.... Storytelling to enhance teaching and learning. Canadian Journal of Dietetic Practice and Research.

[bb0120] Metcalfe A., Haydon J., Bennett C., Farndon P. (2007). Midwives’ views of the importance of genetics and their confidence with genetic activities in clinical practice: implications for the delivery of genetics education. Journal of Clinical Nursing.

[bb0125] Schwartz M., Abbott A. (2007). Storytelling: a clinical application for undergraduate nursing students. Nurse Education in Practice.

[bb0130] Skirton H., Murakami K., Tsujino K., Kutsunugi S., Turale S. (2010). Genetic competence of midwives in the UK and Japan. Nursing and Health Sciences.

[bb0135] Tonkin, E., Calzone, K., Jenkins, J., Lea, D., Prows, C., 2011. Genomic education resources for nursing faculty. Journal of Nursing Scholarship 43 (4), 330–340. 10.1111/j.1547-5069.2011.01415.x 22034967

[bb0140] Wilcock P.M., Brown G.C.S., Bateson J., Carver J., Machin S. (2003). Using patient stories to inspire quality improvement within the NHS modernization agency collaborative programme. Journal of Clinical Nursing.

[bb0145] Ziebland S., Herxheimer A. (2008). How patients’ experiences contribute to decision making: illustrations from DIPEx (personal experiences of health and illness). Journal of Nursing Management.

